# Digital Microfluidic Processing of Mammalian Embryos for Vitrification

**DOI:** 10.1371/journal.pone.0108128

**Published:** 2014-09-24

**Authors:** Derek G. Pyne, Jun Liu, Mohamed Abdelgawad, Yu Sun

**Affiliations:** 1 Department of Mechanical and Industrial Engineering, University of Toronto, Toronto, Ontario, Canada; 2 Department of Mechanical Engineering, Assiut University, Assiut, Egypt; University of Illinois at Chicago, United States of America

## Abstract

Cryopreservation is a key technology in biology and clinical practice. This paper presents a digital microfluidic device that automates sample preparation for mammalian embryo vitrification. Individual micro droplets manipulated on the microfluidic device were used as micro-vessels to transport a single mouse embryo through a complete vitrification procedure. Advantages of this approach, compared to manual operation and channel-based microfluidic vitrification, include automated operation, cryoprotectant concentration gradient generation, and feasibility of loading and retrieval of embryos.

## Introduction

In 1983, the first successful pregnancy following cryopreservation was reported [1]. Three decades later, stem cells [2], sperms [3], and embryos [4] are now routinely frozen and preserved for use at a later time. Patients who undergo therapeutic procedures that can place their fertility at risk, such as chemotherapy, have the option of preserving their reproductive cells (sperms or oocytes/embryos) for future use through in vitro fertilization techniques (IVF) [5]–[8]. Furthermore, extra fertilized embryos after an IVF procedure can also be frozen for use at a later time. The length of time an embryo is frozen has been shown not to have a significant impact on clinical pregnancy, miscarriage, implantation, or live birth [9].

The two commonly used cryopreservation techniques for freezing embryos are the slow freezing method and the vitrification method. Both techniques aim to minimize cell damage caused by freezing that is largely due to the formation of intracellular ice crystals [10]. Conventionally, cells are frozen through the slow freezing method where cells are placed in a large freezer that can accurately control the freezing rate down to liquid nitrogen temperatures, with low concentrations of cryoprotectants [11]. During slow freezing extracellular water freezes away from the embryo, using a seeding technique, which creates an osmotic gradient that draws water out of the cell until it finally freezes without the formation of intracellular ice crystals [12]. This procedure requires sophisticated equipment to control the freezing rate, which ranges between 0.3 and 1.0°C/min, and produces a relatively poor survivability rate [13].

On the other hand, vitrification offers an alternative approach in which cells are frozen at extremely high rates, usually by directly plunging the sample into liquid nitrogen, after bathing them in a sequence of high concentration cryoprotectants [14]. Vitrification reduces intracellular ice formation, which is the primary cause of cell death, by freezing the sample in a glass-like state before the molecules have a chance to form crystal structures. This results in a higher cell survival rate after thawing compared to conventional slow freezing without the need for a seeding procedure or a programmable freezer [13], [15]. However, vitrification requires precise washing sequences and timings in each cryoprotectant medium since higher concentrations are used and there are risks of toxicity if overexposed. The process is expensive in terms of technical skills required. In IVF clinics, processing an embryo/oocyte in cryoprotectant medium typically costs a highly skilled embryologist 10 to 15 minutes.

**Figure 1:**
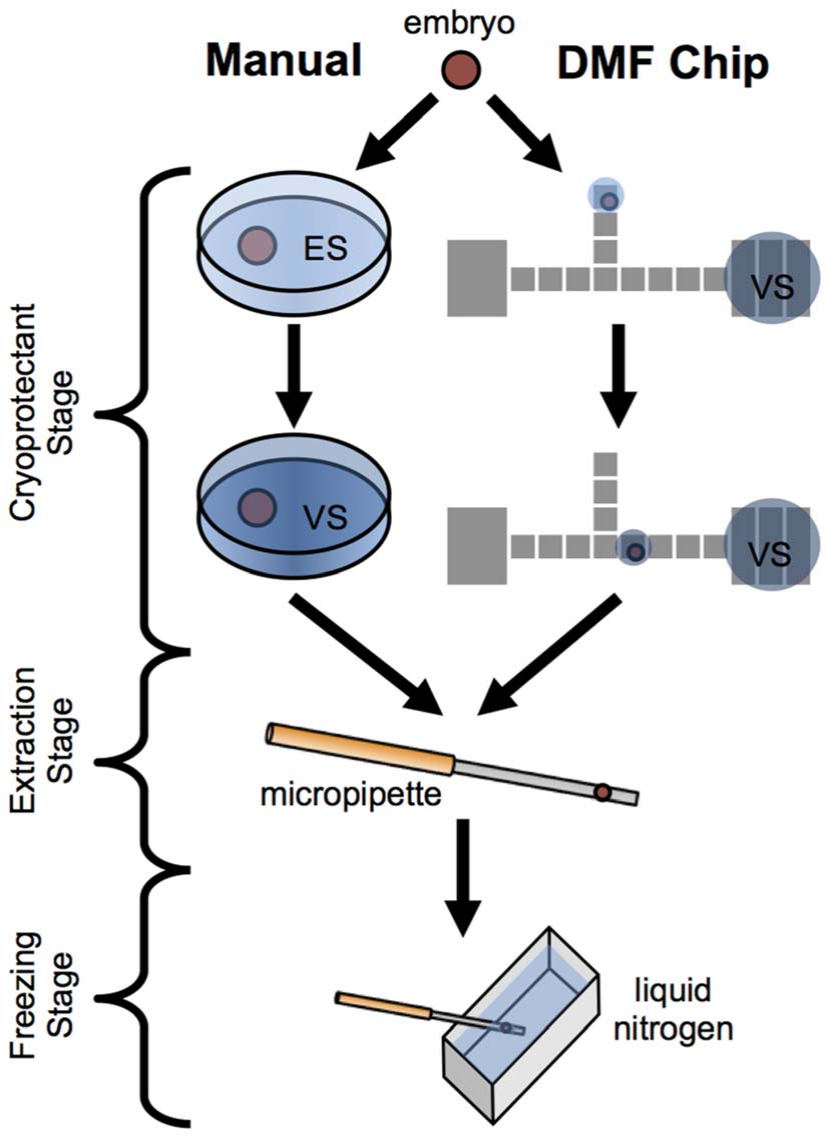
Manual and digital microfluidic vitrification workflow. Schematic showing differences between manual vitrification approach, which requires manual pipetting between cryoprotectant mediums, and the digital microfluidic (DMF) approach, which moves the embryo between mediums on chip. The chip automates the high skill portion of the procedure providing labor cost savings and opportunities for parallel processing.

Digital microfluidics, which enables the manipulation of liquid droplets over an array of electrodes [16], is a useful tool for sequential sample processing and has been used in many biological applications such as PCR, cell culture, and immunoassays [17]–[19]. It was also used for testing cryoprotectant concentrations in slow-freezing cryopreservation [20]. Given its capabilities, digital microfluidics is well poised to automate embryo processing for embryo vitrification. The key to automating the vitrification process is to replicate the washing and timing steps of a given protocol while also keeping complete control of the embryo. Droplets on the digital microfluidic platform can act as micro-vessels to move an embryo and subject it to a series of cyroprotectants of different concentrations, as required by IVF vitrification protocols (Figure 1).

Here we developed digital microfluidic devices, for the first time, to automate embryo preparation for the vitrification procedure, lowering the high labour cost and ultimately helping further spread the use of vitrification in IVF clinics. Although vitrification was demonstrated inside microchannels [21]–[23], the device reported in this paper does not undesirably ‘park’ the embryo to a confined area, which could expose the embryo less uniformly to cryoprotectant medium and has less intricacy of embryo introduction and retrieval onto and from the device. By keeping the embryo in a single droplet, as opposed to microchannels or wells, we are also able to constantly track and control the embryo's locations within the field of view of our imaging system throughout the procedure to avoid cell loss.

## Materials and Methods

### Materials

All mouse experiments were performed in accordance with Canadian Council on Animal Care (CCAC) guidelines for Use of Animals in Research and Laboratory Animal Care under protocols (permit or protocol #:AUP0015) approved by the animal care committees of Toronto Centre for Phenogenomics (TCP). Mouse embryos were gathered from the Canadian Mouse Mutant Repository (CMMR; Toronto, ON). Embryos were produced by superovulating a female and were gathered 2.5 days past conception, which corresponds to most embryos being in the 8-cell stage. Vitrification solution (VS) typically contains antifreezing agents or cryoprotectants, such as dimethyl sulfoxide (DMSO), some small molecular sized glycols (e.g., ethylene glycol), or sucrose [24]. A combination of DMSO and sucrose was used in this work according to the protocol provided by CMMR. The vitrification solution (VS) was made by diluting DMSO in serum-free KSOM medium (EMD Millipore, Billerica, US) at 33% concentration, with 1.0 M sucrose. The equilibrium solution (ES) was at half concentration of VS (i.e., 16.5% DMSO+0.5 M sucrose). VS was preloaded on the DMF chip before each experiment. The first mixing step, which mixes VS with embryo culture medium (i.e., serum-free KSOM), generates the equilibrium solution ES.

### Device construction

Devices were fabricated in the cleanroom facilities of the Toronto Nanofabrication Centre (TNFC). Pre-coated chromium glass slides (Deposition Research Labs Inc., MO) were first primed with Hexamethyldisilazane (HMDS) P-20 (Shin-Etsu MicroSi, Phoenix, AZ) by spin coating (3,000 rpm, 30 sec) before spin coating Shipley S1811 photoresist (3000 rpm, 30 sec). Substrates were then baked to remove solvents (115°C, 2 min) and UV exposed through a transparency mask for 10 sec. Substrates were next developed in MF-321 (2 min), hard baked (115°C, 1 min), etched with CR-4 (2 min) and then photoresist was removed with AZ-300T stripper (15 min in ultrasonic bath). Electrodes (1 mm×1 mm) were separated by a 20 µm gap.

**Figure 2:**
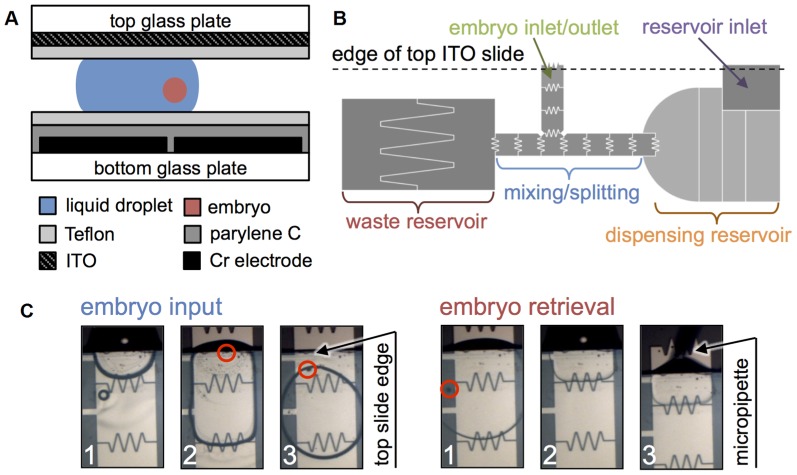
Key device design elements and fabrication layers. (A) Device structure and fabrication. Electrodes (1 mm×1 mm) were separated by a 20 µm gap. (B) Regions for vitrification medium dispensing and for embryo loading/extraction. The top ITO slide is placed on the device in a manner that exposes portions of the top electrodes in the dispensing reservoir and exposes portions of the leg of the T-shaped electrodes, for medium and embryo loading, respectively. (C) Embryo is input and extracted by actuating electrodes at edge of top glass slide.

A 2 µm thick dielectric layer of Parylene C was then deposited. Lastly, a hydrophobic coating of Teflon AF 1600 (Dupont, Mississauga, ON) was spin coated on the device (1% w/w in Fluorinert FC-40, 2000 rpm, 1 min) and baked (160°C, 10 min). A second glass slide coated with un-patterned ITO (Deposition Research Labs Inc., MO) and Teflon AF was used as the ground electrode. Finally, the devices (Figure 2(a)) were assembled by placing the top ITO slide over the patterned device using two pieces of double-sided tape as a spacer. This produces a gap of approximately 100 µm between the top and bottom structures.

### Device design

Voltages applied to actuate droplets were 55–75 Vrms at 15 kHz. Cyroprotectant droplets were actuated inside silicone oil (2.0 cSt, Gelest Inc., Morrisville, PA) to reduce friction and evaporation. Different regions of the device were designed to achieve the general digital microfluidic fucntions of transporting, mixing, dispensing, and splitting droplets. A large reservoir was used to hold and dispense the high concentration cryoprotectant medium, as shown in Figure 2(b). This reservoir was split up into many sections to handle variations in liquid volume in the reservoirs as droplets are dispensed during the vitrification protocol. The second reservoir was used as a waste reservoir and, thus, was split into two large electrodes only. A central inverted T-shaped array of electrodes was used for droplet transport, mixing, and splitting. Electrodes in this array were interdigitated to allow droplet overlap with adjacent electrode and increase electrodynamic forces applied on droplets. Top electrode in the leg of the T-shape was an input/output region where half of the electrode was exposed out of the ITO slide to enable embryo loading.

### Embryo loading and retrieval

As shown in Figure 2(c), to input an embryo, a small droplet (200 nL) containing the embryo was pipetted onto the loading electrode and then actuated into the device, as described in [25]. This technique minimized exposure of the embryo to outside air. Extraction of the embryo was completed in the opposite manner by transporting the embryo-containing droplet to the edge of the device and retrieving it by a micropipette. Once the embryo was extracted from the device, it was directly frozen in the micropipette inside liquid nitrogen.

### Cryoprotectant mixing

**Figure 3:**
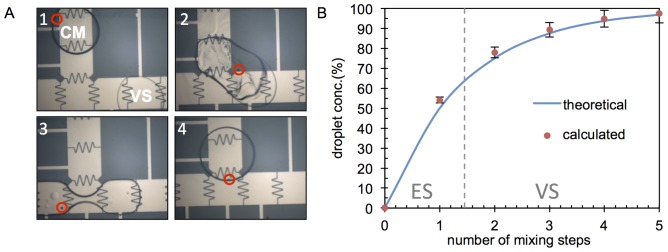
Droplet mixing protocol and resulting concentration profile. (A) 1: Embryo (red circle) contained in culture medium (CM) droplet. 2: Embryo droplet mixed with VS droplet. 3: Droplet split into two droplets (left contains embryo). 4: Droplet containing embryo is kept and other droplet is sent to waste. Process is repeated to increase VS concentration. (B) Mixing profile showing the generation of ES medium and VS medium. Droplet volumes were calculated by imaging droplet boundaries and modeling as cylinders. Concentrations were then calculated using these volumes before and after each mixing step.

An embryo is input into the device in a small droplet of embryo culture medium, and 100% cryoprotectant is input into the device in larger volumes (‘reservoir inlet’ in Figure 2(b)). The cryoprotectant bathing procedure is then performed through a serial mixing/splitting process (see Figure 3). This is accomplished by mixing the embryo-containing droplet with a vitrification solution droplet (VS), thus increasing the concentration of cryoprotectant around the embryo. The resulting droplet is then split into two daughter droplets with the one containing the embryo identified and kept, while the other droplet is moved to the waste reservoir (see Video S1).

After the first mixing step the droplet reaches 50% cryoprotectant concentration (i.e., equilibrium solution or ES), the embryo is kept in the ES droplet as long as the specific protocol requires. Then the cryoprotectant concentration is increased again by droplet mixing and splitting. Contrary to its state in ES medium, embryo volume sharply decreases in VS medium and does not recover. The overall mixing profile generated with a single dispensing reservoir is 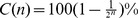,where 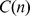 is the concentration of the droplet, and *n* is the number of mixing steps.

This protocol mimics a typical two-step human embryo/oocyte protocol. However, mouse embryos are typically frozen with only a single step protocol where the embryo is directly transferred to VS medium and then frozen. On our device, this corresponds to simply removing the waiting time in the ES medium step. Both protocols were performed; however, the results presented were done with the mouse embryo timings to follow the protocol provided by CMMR.

After complete transfer of the embryo into the VS medium, the droplet containing the embryo is moved toward the edge to be collected by a micropipette (Figure 2(c)), and then plunged into liquid nitrogen. Contrary to conventional vitrification protocols and manual operation, which subject embryos to sudden changes in medium concentration, the digital microfluidic approach gradually increases the VS medium concentration, (Figure 3), which is generally accepted by IVF practitioners to be more benign to embryos due to lower osmotic stress [26].

### Thawing

To verify success of the vitrification process using digital microfluidics, embryos vitrified on device were thawed back and confirmed to have recovered in volume and have healthy morphology. Embryos were thawed by plunging in a bath of culture medium with 1.0 M of sucrose, which helps draw the cryoprotectants out of the embryo to minimize toxicity. The embryo is left in this bath for approximately 10 minutes over which its volume slowly increases back to its original size. After this point the embryo is transferred to culture medium without sucrose and is returned to the incubator. The same thawing procedure was used for manual and digital microfluidic operation.

## Results and Discussion

**Figure 4:**
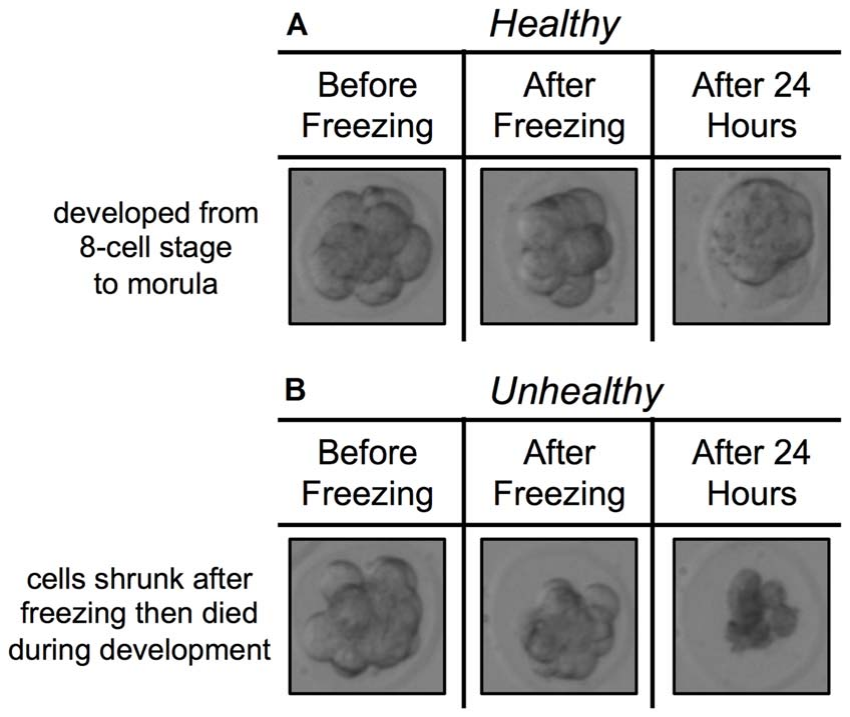
Healthy and unhealthy embryo morphology before and after freezing, and after 24 hours culturing. (A) Healthy and (B) unhealthy examples showing morphology changes before and after freezing. Survival rate was determined on the basis of embryo morphology after thawing vitrified embryos. Cell leakage, abnormal shapes, and membrane damage, as commonly used in vitrification studies, were counted as failure cases. Development rate was quantified by the embryo stage reached after culturing. For instance, the 8-cell stage embryo shown in (A) after vitrification, thawing, and culturing successfully developed to the blastocyst stage.

Survival rate and development rate were used to evaluate the performance of digital microfluidic vitrification. Survivability was measured by examining the morphology of the embryo before and after freezing, as commonly performed in the literature (e.g., [27]). Embryos were considered unhealthy if they had an abnormal shape, membrane damage, leakage of cellular content or degeneration of their cytoplasm [28]. The development rate was determined by culturing survived embryos for an additional 24 hours after thawing (Figure 4). If the cell number within the embryo increased or it developed to the blastocyst stage, it was counted as developed. Control samples of non-vitrified embryos were also cultured to identify the base development rate of the mouse embryo population. Only embryos morphologically judged to be healthy were used for either manual or digital microfluidic testing. Only embryos that had healthy morphology after freezing were cultured following similar procedures to other vitrification studies [29].

**Figure 5:**
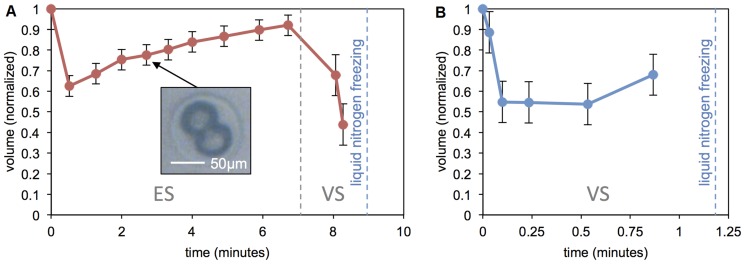
Embryo cell volume monitoring. Mouse embryo cell volume change measured on chip. (A) Vitrified using a two-step human embryo vitrification protocol (Irvine in Figure 6 with the addition of sucrose in the ES stage). (B) Vitrified using a one-step mouse embryo vitrification protocol (CMMR in Figure 6). Snapshots from recorded videos, at instances when the droplet was not moving, were used to measure volume. Volumes were calculated by modeling cells as spheres and were normalized to initial volume (this could result in errors as cells may collapse or flatten instead of shrinking symmetrically [32]. The initial volume dip in the human protocol matches the volume dip over the mouse protocol. For this experiment of volume measurement, 2-cell embryos were used to simplify image analysis. Error bars in (B) are relatively large because for this vitrification protocol, droplets are required to move quickly, which did not leave sufficient time to switch to higher microscope magnification for imaging on our digital microfluidic platform.

Table 1 summarizes the experimental results, showing comparable survival and development rates between manual and automated processing. Additionally, since the embryos are constantly imaged on chip, its volume can be measured throughout the procedure and used to measure the quality of both the embryo and the protocol (Figure 5). Embryo relative volume response to the VS of different concentrations is an interesting and complex issue for developing new vitrification protocols [30], [31]. The measurement of cell volume is difficult because embryos may collapse or flatten in VS instead of shrinking symmetrically [32].

**Table 1:** Summary of vitrification results.

	Survival Rate	Development Rate
control (non-vitrified)	100% (14/14)	93% (13/14)
manual	73% (11/15)	91% (10/11)
DMF Chip	77% (10/13)	90% (9/10)

Previous studies of embryo vitrification (4–16 cell stages) reported survival rates in the range of 80–100% [4]. Based on the limited sample size, vitrification using digital microfluidics in our work produced a survival rate of 77%, and our own manual vitrification trials resulted in a survival rate of 73%. These lower survival rates, compared to the results in the literature, can be mainly attributed to our use of a micropipette (vs. vitrification straw) inside liquid nitrogen. The micropipette tip is a standard plastic pipette for embryo manipulation and had an inner diameter of 125 µm (The STRIPPER micropipetter, Origio). Much research has gone into developing different mechanical structures (e.g., straw-type carriers [33], cryotube [34], Cryotop [35], and mesh-type carriers [36]) to increase the heat transfer rate [12]. Using these devices in liquid nitrogen requires the transfer of a processed embryo onto the device (e.g., vitrification straw) with minimal liquid volume remained on the vitrification straw. Since this embryo transfer process is not within the capability of our present microfluidic device and would introduce an extra manipulation step by hand, this work focused on proving the feasibility of using digital microfluidics for embryo processing. Therefore, the microfluidically processed embryos were directly frozen inside the micropipette tip in our experiments.

We are aware that using micropipette tips in liquid nitrogen was not ideal, which should have negatively affected the survival rate; however, since it was held constant between manual and DMF trials, its affect should be systematic. Additionally, the development rate, which was measured using embryos that survived freezing as commonly practiced in the literature, was high and comparable with other vitrification studies. This development rate measurement desirably removes the effect of our limited manual embryo handling skills and shows the potential of our microfluidic device for automated processing of embryos for vitrification.

**Figure 6:**
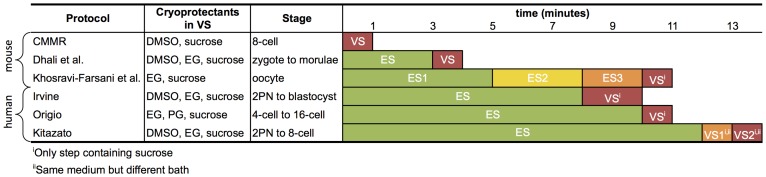
Comparisons of mouse and human vitrification protocols [28], [29], [42]–[47]. For those protocols that specify a timing range, the average value is used.

Due to its programmability, one key benefit to the digital microfluidic approach is the ability to implement/test a number of vitrification protocols for efficacy comparisons. Figure 6 lists example human and mouse vitrification protocols, all using different cryoprotectants, number of mediums, and timings. However, at their core, all of these protocols involve the controlled increase in cryoprotectant concentration over a given period of time with the initial equilibrium solution typically containing half the cryoprotectant concentration as the full strength vitrification solution. This is convenient for DMF device design as it always produces a 50% concentration after the first mixing (neglecting the time required to homogenize concentration of the resulting droplet which is in the order of 2∼3 seconds when the droplet is rolled over a few electrodes to enhance mixing). This means that a typically two-step protocol involving an initial 50% concentration ES step, following by a short 100% VS step, can be easily realized by following the mixing curve shown in Figure 3 with a pause after the first mixing step to allow the embryo to reach equilibrium.

**Figure 7:**
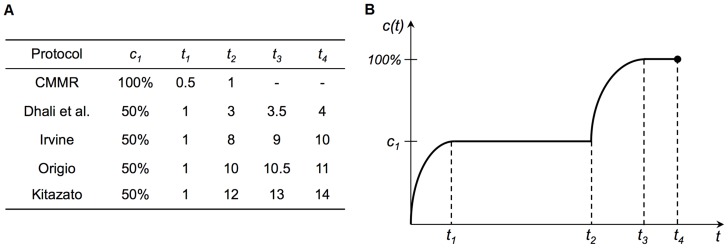
Vitrification protocol implementation on digital microfluidic chip. Implementation of common vitrification protocols on a digital microfluidic chip with a single dispensing reservoir. Timings and concentrations are shown in (A), and the generalized mixing curve is shown in (B).

Figure 7 shows how most two-step protocols follow the same mixing curve, only differing by their timings. This shows that although the protocols in Figure 6 involve significantly different amounts of manual manipulation, when performed on the digital microfluidic chip, they follow the same mixing procedure with the only difference lying in their timings. For our current trials, a mouse protocol provided by CMMR was used which involved a single mixing step. This allowed us to better conduct our manual trials as it required less manipulation; however, more complicated protocols can be readily performed by adding more dispensing reservoirs on chip and filling them with lower concentrations of cryoprotectant. This would allow for a high number of producible concentrations, especially in the low concentration range. Multiple reservoirs could also be used to implement protocols using different cryoprotectant compositions throughout their procedure [37].

One limitation of the digital microfluidic platform was handling culture mediums containing high serum concentrations. Serum contains a mixture of proteins that can adsorb on the Teflon coated surfaces of the device, eventually accumulating to the point that the surface becomes hydrophilic, making droplets immovable [38]. Some strategies have been developed to help overcome this problem, such as the use of Pluronic additives [39], silicone oil baths [40], and superhydrophobic surfaces [41]. Pluronic additives were avoided in our work as embryos are highly sensitive to additives. Superhydrophobic surfaces were also avoided as they require significant additional fabrication efforts. A silicone oil bath was used which did increase droplet movability; however, this approach did not work with sufficient effectiveness for conventional embryo culturing mediums that contain high serum concentrations. Therefore, in this work, a serum free culture medium was chosen for this proof-of-principle study. In further work, we will explore droplet movability improvement for serum-containing culture medium and the automation of transferring embryos onto vitrification devices (e.g., straw or Cryotop).

## Conclusion

This paper described a digital microfluidic device that shows feasibility to perform automated embryo processing for vitrification applications. The results demonstrated that the embryo survival and development rate achieved by using the automated approach are comparable to manual operation, based on the limited sample size tested. Advantages of this approach, compared to manual operation and channel-based microfluidic vitrification, include automated operation, cryoprotectant concentration gradient generation, and feasibility of loading and retrieval of embryos. The device permits one to readily modify/test vitrification protocols with reduction in labor costs. Further development could possibly facilitate new vitrification protocol development and clinical IVF practice.

## Supporting Information

Video S1 **Automated vitrification of mouse embryo using digital microfluidic chip.**
(MOV)
